# Photosynthetic efficiency, growth and secondary metabolism of common buckwheat (*Fagopyrum esculentum* Moench) in different controlled-environment production systems

**DOI:** 10.1038/s41598-021-04134-6

**Published:** 2022-01-07

**Authors:** Marta Hornyák, Michał Dziurka, Monika Kula-Maximenko, Jakub Pastuszak, Anna Szczerba, Marek Szklarczyk, Agnieszka Płażek

**Affiliations:** 1 Department of Physiology, Plant Breeding and Seed Production, University of Agriculture, Podłużna 3, 30-239 Kraków, Poland; 2 W. Szafer Institute of Botany, Polish Academy of Sciences, Lubicz 46, 31-512 Kraków, Poland; 3 Institute of Plant Physiology, Polish Academy of Sciences, Niezapominajek 21, 30-239 Kraków, Poland; 4 Faculty of Biotechnology and Horticulture, University of Agriculture, 29 Listopada 54, 31-425 Kraków, Poland

**Keywords:** Plant physiology, Plant breeding

## Abstract

Light-emitting diodes (LEDs) and high-pressure sodium lamps (HPS) are among the most commonly used light sources for plant cultivation. The objective of this study was to evaluate the effect of two controlled-environment production systems differing in light sources on growth, photosynthetic activity, and secondary metabolism of common buckwheat. We hypothesized that LED light with the majority of red and blue waves would increase physiological and biochemical parameters compared to sunlight supplemented with HPS lamps. The experiment was performed in a phytotronic chamber (LEDs) and in a greenhouse (solar radiation supplemented with HPS lamps as a control). The effects were analyzed at the flowering phase with biometric measurements, leaf chlorophyll index, the kinetics of chlorophyll *a* fluorescence, content of soluble carbohydrates and phenolics in the leaves. Applied LED light decreased the biomass but stimulated the production of phenolics compared to control plants. In control plants, a positive correlation between flavonoid content and energy dissipation from photosystem II (DI_o_/CS_m_) was found, while in plants under LEDs total pool of phenolic content correlated with this parameter and the quantum yield of electron transport (φ Ro and ψ Ro) was lower than that of the control, probably affecting buckwheat biomass.

## Introduction

Among primary environmental factors, light is probably the most important one affecting the growth and development of plants^1^. It is essential for germinating, seedling development, generative phase, photosynthesis productivity, and it is a significant signal to mediate substance metabolism for plant tolerance to environmental fluctuations^2,3^. Chlorophylls are the primary photosynthetic pigments that absorb primarily red and blue light, and so these wave ranges are thought to support plant development to the most significant degree. Blue light photoreceptors (cryptochromes and phototropins) and red/ far-red light receptors (phytochromes) monitor light spectra to control plant phenotype by regulating their growth and development. Another group of pigments, carotenoids, absorb violet and blue-green light. The examples of developmental responses include leaf expansion area, stem length, stomata opening, flowering, and phototropism^1,4^. Plants exposed to adverse fluctuations of environmental conditions exhibit photosynthesis disturbances. Analysis of chlorophyll *a* fluorescence (ChlF) is commonly used to study the photochemical efficiency of leaves and plant physiological conditions. It is a non-invasive and powerful tool in ecological and environmental studies of plant response to stress factors^5^. Light also affects the synthesis of some plant metabolites such as sugars, the primary photosynthetic assimilates, or phenolic compounds^4,6^, and regulates their secondary metabolism through induction of phenolic biosynthesis by affecting the activity of phenylalanine ammonia-lyase (PAL), which is the first enzyme in the phenylpropanoid pathway^7,8^. Phenylpropanoids originate from cinnamic acid, formed from phenylalanine in a reaction catalyzed by PAL, the offshoot point among primary (shikimic acid pathway) and secondary (phenylalanine pathway) metabolism^9^. Plants are potential sources of bioactive compounds such as phenolic compounds, demonstrating mainly antioxidant ability. Phenolics are recognized as pro-healthy components stored mainly in plant leaves. Secondary metabolites (such as phenolics) are accumulated often under stress conditions^10^. Sugars are significant regulators of metabolism; growth and their accumulation could be an indicator of photosynthetic efficiency^11^. The synthesis of phenolic compounds requires an extensive energy input, and therefore it depends on the accumulation of soluble sugars in the cells. To gain optimal photosynthetic productivity, each leaf within the plant canopy needs to adapt to fluctuations in light intensity and quality rapidly^12^. For example, Lin et al.^13^ proved that Red Blue White LED lights increased soluble sugar content and their accumulation in lettuce leaves. It positively affected their growth, development, and nutrition. It was also observed that high light intensity treatment supplemented with blue light reduced the quantum yield of photosystem II (PSII) and increased the accumulation of, i.e., pigment and phenolic content in *L. sativa*^14^. Phenolics affect various physiological processes related to plant growth and development, such as seed germination, cell division, flower development, and synthesis of photosynthetic pigments^15,16^. Various phenolic compounds have essential roles in human and animal functioning due to their practical, biological, and pharmacological effects, mainly because they protect the cell from oxidative stress. Gallic acid participates in cell apoptosis in different human diseases^17^; ferulic acid was involved in innovative mechanisms related to Alzheimer’s disease^18^. Chlorogenic acid demonstrates pharmacological properties, for example, antioxidant activity, antibacterial, antiviral and antimicrobial attributes^19^.

Controlled-environment plant production systems are widely used worldwide to produce plant materials or products of a quality that cannot be obtained in the natural environment. The primary environmental parameter controlled is temperature. However, environment control can contain other factors such as carbon dioxide levels, relative humidity, water, pest control, plant nutrients, and light. Therefore, manipulating light quality, spectrum, and light intensity to obtain better plant growth and quality has become a popular research object in recent years^20^. Artificial light can increase crop yield and nutritional value, especially during the late autumn and winter in greenhouse cultivation in northern latitudes^2–4^. In a greenhouse, the lighting conditions are difficult to control due to the change in light intensity depending on the season, time of day, and degree of cloudiness. On the other hand, daylight from dawn to dusk is the most natural factor for plant growth and development. The studies carried out in phytotronic chambers are, however, justified. Plant cultivation indoor allows to provide plants with more stable temperature, soil hydration, and light conditions specific to the species, and may also be important for urban agriculture. In the greenhouse or phytotronic conditions, more frequent crops can also be harvested, in some cases 2–3 times a year, and the growing conditions can be selected to obtain better plant quality in terms of the content of health-promoting compounds^21^. The most widespread light sources used for controlled environmental agriculture during the last decades are high-pressure sodium (HPS) lamps^6,22^. This kind of light provides high photon flux emission and long operational life but tends to use a large amount of electricity and radiates much heat, which leads to energy waste. Moreover, often their most intensive green and yellow bands of the spectrum are not optimal for photosynthesis^4,23^. Chlorophylls cannot absorb much light in the 450–550 nm spectrum, i.e., maximum sunlight intensity on Earth. However, this is the exact waveband where the carotenoids are the most efficient at light-absorbing^24^. light-emitting diodes (LEDs) proposed as an alternative light source have distinct advantages, such as low radiant heat output, reduced energy consumption, and composing their spectra to optimize photosynthesis and regulate plant growth and development^3,6^. However, LEDs should be compatible with the photosynthesis requirements of grown plants due to the specific role of each waveband of light. The study of monochromatic light creates a great opportunity to understand basic plant physiology processes. The current state of knowledge determines that blue and red light, are efficiently absorbed close to the surface while a green light contributes to deeper layers of the leaf and on the lower canopy level. It means that green light may drive photosynthesis in areas where other wavelengths are in a limited amount. The UV and far-red parts of the sunlight spectrum are important in defining photomorphogenesis^21^.

Buckwheat (*Fagopyrum esculentum* Moench) belongs to the *Polygonaceae* family, and it is considered a pseudo-cereal that constitutes a valuable food source in several regions of the world. Nowadays, it is becoming more popular because of its excellent nutritional qualities and gluten-free seeds. Its grains are rich in compounds such as polyphenols, lipids, dietary fiber, polysaccharides, and amino acids^25,26^. Therefore, this species is the subject of intensive research studies all over the world. Increasing yield productivity demands a better understanding of the environmental parameters that determine seed yield and seed composition. An increase in the assimilate synthesis is one of the major factors in plant productivity growth. Thus far, the intensity of buckwheat photosynthesis was studied, e.g., by Amelin et al.^27^. They concluded that the buckwheat cultivars with determined growth habit (*det* mutation) showed a higher photosynthesis rate at the grain filling stage than those with indeterminate growth habit. However, there are no data on the light quality impact on the growth and development of common buckwheat.

Our previous experiments found that common buckwheat plants sown and cultivated under sole HPS light at optimal temperature and humidity displayed abnormalities in morphology at the cotyledon stage and stopped growing. That disturbed ongoing experiments. However, the plants sown and grown to the cotyledon stage under solar light supplemented with HPS developed properly throughout further vegetative and generative stages^28^. The plants under both treatments grew at 25 + 2 °C/22 ± 2 °C day/night, with 55–60% humidity and under a 16 h photoperiod. It encouraged us to investigate this phenomenon in the current study. The hypothesis was that LED lamps emitting mainly blue and red waves, absorbed mainly by chlorophylls, can significantly influence common buckwheat's physiological and biochemical parameters. The objective of the present study was to evaluate the effect of two controlled-environment production systems differing in light sources on growth, photosynthetic activity, and bioactive compounds content in common buckwheat plants.

## Results

### Plant growth measurements

**Figure 1:**
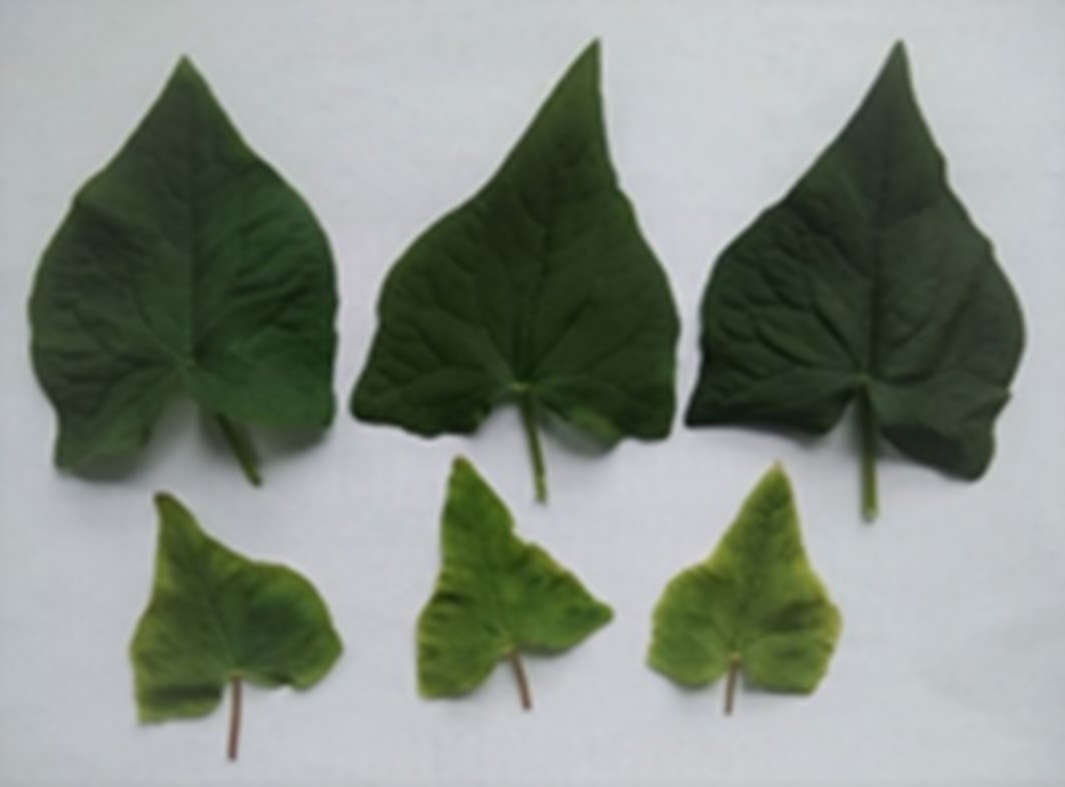
Differences in the leaf area in the plants grown in greenhouse conditions under solar light supplemented with HPS Agro Philips lamps (upper row) and the plants grown in phytotron chambers under LED light (bottom row). All presented leaves were collected at the same time from 8-week-old plants. The sample was the third, fully developed leaf in order from the top inflorescence.

Common buckwheat plants grown under LED light were more compact (smaller leaf area and more internodes) and showed lower main stem height than the plants grown in the glasshouse with solar light supplemented with HPS lamps (Table 1). The number of internodes was not significantly different between both treatments. The plants grown under LED light showed lower fresh weight (FW) and dry weight (DW) of the aboveground parts than those grown in the glasshouse conditions. The leaf area was four times greater, and the FW of the leaf was three times higher for the plants grown under solar light supplemented with HPS lamps than those grown under LED light (Table 1; Fig. 1).

**Table 1:** Main stem height, fresh (FW), and dry weight (DW) of the aboveground parts (stems with leaves) as well as leaf area of common buckwheat cv. ‘Panda’ grown under different controlled-environment production systems.

Light	Main stem height [cm]	No. of the main stem internodes	Aboveground parts FW [g]	Aboveground parts DW [g]	The third leaf area [cm^2^]	The third leaf FW [g]
Control	86.1 ± 1.7	9.7 ± 0.4	14.765 ± 1.2	1.875 ± 0.1	36.0 ± 2.5	0.6 ± 0.06
LED	72.5 ± 2.4**	10.4 ± 0.4	11.014 ± 0.9*	1.478 ± 0.2***	9.5 ± 0.8***	0.2 ± 0.02***

Control—solar light supplemented with HPS (High-Pressure Sodium) lamps; LED (Light-Emitting Diodes). Values represent means (*n* = 10) ± SE. Values marked with stars differ from the control significantly according to the Student’s *t* test: *p < 0.05; **p < 0.01; ***p < 0.001.

### ChlF (chlorophyll *a* fluorescence), chlorophyll index, total soluble carbohydrate, phenolic, and flavonoid content

The overall performance index of PSII (PI) was higher for plants grown under control conditions than in those grown in the phytotron chamber (Table 2). The values of energy used for electron transport (ET_o_/CS_m_), and the number of active reaction centers (RC/CS_m_) were not significantly different between plants grown under control light and LED light. Under LED light, the energy absorption (ABS/CS_m_), excitation energy trapped in PSII reaction centers (TR_o_/CS_m_), and energy dissipated from PSII (DI_o_/CS_m_) were higher than in control. The probability that a trapped exciton moves an electron into the electron transport chain beyond Q_A_^−^ (ψ Ro), as well as the quantum yield of electron transport from Q_A_^−^ to the PSI end electron acceptors (φ Ro) were higher in the plants grown under solar light supplemented with HPS lamps than under LED light. The values of δRo, denoting the efficiency with which an electron can move from the reduced intersystem of electron acceptors to the PSI end electron acceptors, did not differ between the treatments. Detailed, raw data can be found in the supplementary material (Supplementary Table S1).

**Table 2:** Changes in the kinetics of chlorophyll a fluorescence in common buckwheat plants of cv. ‘Panda’ grown under different controlled-environment production systems.

Light	PI	ABS/CS_m_	TR_o_/CS_m_	ET_o_/CS_m_	DI_o_/CS_m_	RC/CS_m_	ẟ Ro	φ Ro	ψ Ro
Control	2.1 ± 0.2	1492 ± 10	1244 ± 10	594 ± 20	247 ± 3	633 ± 6.3	0.36 ± 0.01	0.14 ± 0.01	0.17 ± 0.01
LED	1.5 ± 0.2***	1591 ± 23***	1305 ± 25*	545 ± 34	286 ± 11***	640 ± 21	0.36 ± 0.02	0.12 ± 0.01*	0.14 ± 0.01***

Control—solar light supplemented with HPS (High-Pressure Sodium) lamps; LED (Light-Emitting Diodes). Values represent means (*n* = 20) ± SE. Values marked with stars differ from the control significantly according to the Student's *t* test: *p < 0.05; **p < 0.01; ***p < 0.001. ABS/CS_m_—energy absorption by antennas, DI_o_/CS_m_—energy dissipation from PSII, ET_o_/CS_m_—the energy used for electron transport, PI—performance index of PSII, RC/CS_m_—number of active reaction centers, TR_o_/CS_m_—excitation energy trapped in PSII, δ Ro—efficiency with which an electron can move from the reduced intersystem of electron acceptors to the PSI end electron acceptors, ψ Ro—probability, at time 0, that a trapped exciton moves an electron into the electron transport chain beyond Q_A_^−^, φ Ro—quantum yield of electron transport from Q_A_^−^ to the PSI end electron acceptors.

Chlorophyll index was more significant in the leaves developed under solar light supported with HPS lamps than in the plants grown under LED light (Table 3). This effect is also shown in Fig. 1. Phenolic and flavonoid content was more significant in the leaves developed under LEDs light than under solar light supplemented with HPS (Table 3).

**Table 3:** Chlorophyll index, the average content of total phenolics^a^ [µmol mg^**–1**^ DW] and total flavonoids^b^ [nmol mg^**–1**^ DW] in leaves of common buckwheat cv. 'Panda' under different controlled-environment production systems.

Light	Chlorophyll index	Phenolic content [µmol mg^–1^ DW]	Total flavonoid content [nmol mg^–1^ DW]
Control	18.9 ± 1.4	0.186 ± 0.015	52.4 ± 2.9
LED	14.5 ± 0.8**	0.449 ± 0.020***	114.4 ± 5.8***

Control—solar light supplemented with HPS (High-Pressure Sodium) lamps; LED (Light-Emitting Diodes). Values represent means (*n* = 20 for chlorophyll index; *n* = 3 for phenolic and total flavonoid content) ± SE. Stars indicate significant difference between means; **p < 0.01, ***p < 0.001 (Student’s *t* test).

^a^µmol gallic acid equivalent (GAE)/mg^**–1**^ DW.

^b^µmol quercetin equivalent (QUE)/mg^**–1**^ DW.

### Individual sugar content (SC)

**Figure 2:**
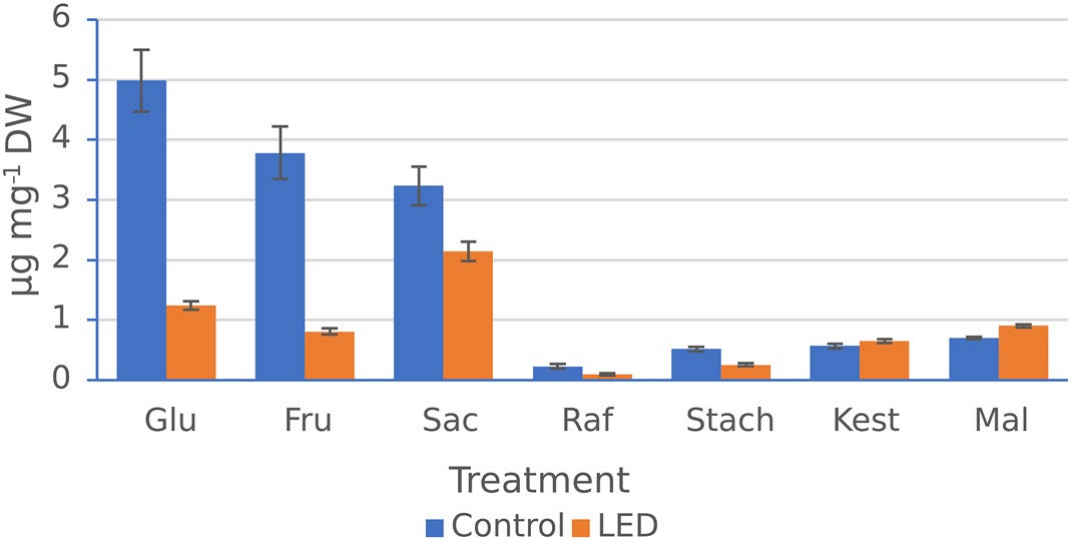
Individual sugar content in leaves of common buckwheat cv. ‘Panda’ under different controlled-environment production systems. Control—solar light supplemented with HPS (High-Pressure Sodium) lamps; LED (Light-Emitting Diodes), Raf—raffinose, Stach—stachyose, Kest—1-kestose, Mal—maltose, Glu—glucose, Fru—fructose, Suc—sucrose. Values represent means (*n* = 3) ± SE. Stars indicate significant difference between means; *p < 0.05, **p < 0.01, ***p < 0.001 (Student's *t* test).

Identification and quantification of sugar content in the leaves of common buckwheat showed significant differences between control and LED treatment (Fig. 2). The individuals studied sugars were significantly lower in the plants grown under LED, except for kestose and maltose. The highest differences were found for glucose (its amount was four times lower), fructose (five times lower), and sucrose (one and half times lower compared to that of the control).

### Phenolic acid content

Plants grown under LED light produced significantly more amounts of the following phenolic acids: gallic, 3,4 dihydroxybenzoic, caffeic, vanillic, chlorogenic, homovanillic, syringic, benzoic, rosmarinic, and cinnamic than those grown under solar light supplemented with HPS lamps (Table 4). Buckwheat grown under solar light supplemented with HPS lamps contained higher *p*-hydroxybenzoic, ferulic, and salicylic acids than the plants treated with LEDs. The content of gentisic and *p*-coumaric acids remained at the same level under both light treatments.

**Table 4:** The average amount of phenolic acids [µg mg^–1^ DW] in common buckwheat cv. 'Panda' grown under different controlled-environment production systems.

Phenolic acid	Control [µg mg^–1^ DW]	LED [µg mg^–1^ DW]
Gallic acid (3,4,5-trihydroxybenzoic acid)	0.044 ± 0.006	0.114 ± 0.012***
3,4-Dihydroxybenzoic acid	0.008 ± 0.001	0.018 ± 0.002**
*p*-Hydroxybenzoic acid	0.004 ± 0.001	0.002 ± 0.0002*
Gentisic acid (2,5-dihydroxybenzoic acid)	0.005 ± 0.001	0.004 ± 0.001
Caffeic acid (3,4-dihydroxycinnamic acid)	0.017 ± 0.002	0.050 ± 0.005***
Vanillic acid (4-hydroxy-3-methoxybenzoic acid)	0.007 ± 0.0007	0.014 ± 0.001**
Chlorogenic acid	9.4 ± 1.4	25.6 ± 2.7***
Homovanillic acid	0.390 ± 0.051	1.080 ± 0.151***
Syringic acid	0.019 ± 0.003	0.061 ± 0.005***
*p*-Coumaric acid	0.018 ± 0.006	0.012 ± 0.003
Ferulic acid	0.015 ± 0.001	0.007 ± 0.0005**
Benzoic acid	0.078 ± 0.017	0.440 ± 0.052***
Sinapic acid	Nd	Nd
Salicylic acid	0.002 ± 0.0002	0.001 ± 0.0001*
Rosmarinic acid	0.009 ± 0.002	0.064 ± 0.008***
Cinnamic acid	0.005 ± 0.0005	0.010 ± 0.0007**

Control—solar light supplemented with HPS (High-Pressure Sodium) lamps; LED (Light-Emitting Diodes). Values represent means (*n* = 3) ± SE. Values marked with stars differ from the control significantly according to the Student’s *t* test: *p < 0.05; **p < 0,01 ***p < 0.001. nd—not detected.

### Correlation analyses

In control plants grown in a greenhouse under sunlight supplemented with HPS lamps, significant correlations were found between the performance index (PI) and the chlorophyll index in the leaves (r = 0.563; *p* < 0.05), with the fresh and dry weight of the aboveground parts (r = 0.537 and r = 0.617; *p* < 0.05, respectively), with the ChlF parameters such as φ Ro and ψ Ro (r = 0.845 and r = 0.823; *p* < 0.05, respectively) (Table 5). It is worth mentioning that the ChlF related to the quantum efficiency φ Ro and ψ Ro correlated with the fresh and dry mass of the aboveground parts. The energy dissipation from PSII (DI_o_/CS_m_) positively correlated with the content of flavonoids (r = 0.639; *p* < 0.05). In plants grown under LED light, PI correlated with the content of some individual sugars: glucose, sucrose, and maltose. In these plants, the DI_o_/CS_m_ parameter correlated with the total phenolic content.

**Table 5:** Pearson coefficients of linear correlation (*p* < 0.05) between selected studied parameters of common buckwheat cv. ‘Panda’ grown under different controlled-environment production systems.

Variable	PI	Chl	FW	DW	DI_o_/CS_m_	φ R_o_	ψ R_o_	Flavonoids
**Control**								
PI	–	0.563	0.537	0.617	–	0.845	0.823	–
Chl	0.563	–	–	–	–	0.705	0.723	–
FW	0.537	–	–	0.959	–	0.680	0.686	–
DW	0.617	–	0.959	–	–	0.639	0.643	–
DI_o_/CSm	–	–	–	–	–	–	–	0.639
φ R_o_	0.845	0.705	0.680	0.639	–	–	0.998	–
ψ R_o_	0.823	0.723	0.686	0.643	–	0.998	–	–
Flavonoids	–	–	–	–	0.639	–	–	–

Variable	PI	Glu	Suc	Mal	DIo/CSm	Phenolics		
**LED**								
PI	–	0.565	0.456	0.744	–	–		
Glu	0.565	–	0.953	0.793	–	–		
Suc	0.456	0.953	–	0.692	–	–		
Mal	0.744	0.793	0.692	–	–	–		
DI_o_/CSm	–	–	–	–	–	0.653		
Phenolics	–	–	–	–	0.653	–		

Control—solar light supplemented with HPS (High-Pressure Sodium) lamps; LED (Light-Emitting Diodes); PI—performance index of PSII, Chl—chlorophyll index, FW—fresh weight, DW—dry weight, DI_o_/CS_m_—energy dissipation from PSII, ψ Ro—probability, at time 0, that a trapped exciton moves an electron into the electron transport chain beyond Q_A_^−^, φ Ro—quantum yield of electron transport from Q_A_^−^ to the PSI end electron acceptors, Glu—glucose, Suc—sucrose, Mal—maltose.

## Discussion

LED application is a proven and viable option of supplemental lighting in plant cultivation under controlled environmental conditions (e. g., Massa et al.^29^). The theoretical maximum efficiency occurs when the total input energy is transformed into the energy of photosynthetically active photons^30,31^. The blue LEDs are 93% efficient, white in 76%, and red in 81%. Therefore, horticultural LED lamps usually provide combinations of red (peak at 660 nm), blue (peak at 450 nm), and white or far-red (peak at 730 nm) emitting diodes. Other wavelengths are available, but they have lower photosynthetic efficiency^30^. Red and blue LED composition is commonly used for plants grown but still tested to intensify the horticultural production. Wojciechowska et al.^32^ reported that the value of performance index (PI) was the greatest in leaves of lamb lettuce (*Valerianella locusta*) under LED lamps compared to natural light. Our study analyzed the effect of sole LED light on various growth and photosynthetic parameters of common buckwheat, while plants grown under solar light supplemented with HPS lamps served as controls in greenhouse conditions. Our previous experiment conducted under solo HPS lamps indicated that the development of buckwheat plants stopped at the cotyledons stage due to inadequate composition of light spectra. A similar effect was observed in the case of winter rape (data not published). Interestingly, cereals grown under the same light spectrum in the same phytotron chamber developed properly (our observation provided in another study; unpublished). This effect suggests that dicotyledonous plants could have different light demands than monocotyledonous ones. The rapid climate changes taking place on Earth in recent years will probably force food producers to increasingly use the cultivation of plants under glasshouse conditions. Cultivation of plants outside the growing season requires artificial plant lighting, and for this purpose, the spectra emitted by different types of lamps should be adjusted to each plant species. In the case of common buckwheat, the use of solo HPS lamps, usually dedicated to greenhouse crops, was unsuccessful. Therefore, in our research, we used LED lamps with the spectrum most effectively in the photosynthesis process.

Lighting the plants in the greenhouse in the morning with HPS lamps slightly changed the spectrum of light reaching the plants. This spectrum was characterized by a lower proportion of blue light by 4%, violet and far-red light by 3%, and an increase in yellow light by 7% and orange light by 6%. It seems, however, that these relatively small changes could not have had a major impact on the studied parameters, taking into account that the HPS lamps were only used for 4 h early in a day when the solar radiation was weaker. The spectrum of sunlight and sunlight supplemented with HPS lamps was diametrically different from the spectrum of LED lamps. The LEDs in the phytotron chamber emitted 53% red light, 25% blue light, 15% orange light, 1% yellow and green light each. The percentage of far-red and violet light was the same as in the greenhouse. The mean intensity of daylight in the morning supplemented with HPS lamps was comparable to the light intensity emitted by LEDs in the phytotron chamber, but the intensity of sunlight at noon was very high and was 1300–1600 µmol m^–2^ s^–1^. It is worth noting that illumination with HPS lamps as well as solar radiation did not cause significant temperature fluctuations due to the air conditioning of the greenhouse chamber.

In our experiment, LEDs did not have the same effect on plant biomass as solar light supplemented with HPS lamps. The main effect of proper plant development and transition from the vegetative to the generative phase was achieved. In the case of plants grown under LEDs, practically all biometric parameters were significantly lower than that of the control. The most significant differences were observed in the leaf area: the leaves of the plants from the phytotronic chamber were almost four times smaller, which was obviously related to their three times less weight than the leaves of the control plants. The increase in biomass is influenced not only by its intensity but also by the spectrum of light^21^. Studies on the effect of monochromatic red light demonstrated that it inhibits the growth of biomass and leaf area while stimulating shoot elongation, number of leaves, and chlorophyll content^33^. It was also interesting that at the more significant amount of blue and red lights, most conducive to photosynthesis, the overall performance index of PSII (PI) was lower than in the plants grown in the greenhouse. In the plants grown in the phytotron chamber, the greater amount of energy dissipated from PSII (DI_o_/CS_m_) could be an effect of greater energy absorption by the reaction center (ABS/CS_m_) than in the plants grown in the greenhouse. The plants grown under LED and solar spectrum with HPS, demonstrated the same number of reaction centers (RC/CS_m_), although different chlorophyll amounts were detected. Hamdani et al.^33^ reported, that in rice leaves blue light compared to white light decreases the maximum efficiency of PSII (F_v_/F_m_), a lower rate of reduction of the excited electronic state of P700, and increases nonphotochemical quenching (NPQ). In turn, Su et al.^34^ observed an increase in photosynthesis rate in cucumber in monochromatic blue light which was associated with higher Rubisco biosynthesis. Hattori et al.^35^ showed that red light reduced stomatal density, stomatal conductance, the content of Rubisco, and decreased photosynthesis rate. In our experiment, we did not study the effect of monochromatic red light, but we should take into consideration the much higher participation of red light in radiation emitted by LEDs compared to sunlight. LEDs emitted twice more red light than blue light. It is possible that the performance index (PI) was significantly lower in plants grown under LEDs than in the glasshouse due to the high participation of red light and lower light intensity. Energy absorbed by reaction centers (RC) in PSII (ABS/CS_m_) was higher in plants grown in the phytotronic chamber than in the glasshouse. This fact could suggest an adaptation of the leaves to long vegetation in lower light intensity^36^. The higher values of TR_o_/CS_m_ and DI_o_/CS_m_ in these plants are the consequence of higher value ABS/CS_m_. In another study, barley leaves grown in sunlight demonstrated a higher CO_2_ assimilation rate and higher values of electron transport rate (ETR) than in the leaves grown in the shade^37^. At the same time, in the leaves growing in the shade a greater degree of energy dissipation was observed, which would confirm our results. Lazár^38^ suggested a decreased size of the pool of PSII and PSI electron carriers between Q_A_^−^ to ferredoxin in the leaves grown in the shadow.

As mentioned above, in plants under the LED lamps energy absorption (ABS/CS_m_) and energy dissipation from PSII (DI_o_/CS_m_) were higher than in plants grown in a greenhouse condition. This is most likely due to the greater amount of blue and red light absorbed mainly through chlorophyll. At the same time, the amount of absorbed energy did not reflect better quantum yield (ψ Ro and φ Ro), which were significantly lower than in plants grown in a greenhouse. A lower value of these parameters may also indicate the activation of cyclic photophosphorylation, in which mainly ATP is produced and NADPH not, which is needed for the reduction of 3-phosphoglyceric acid to 3-phosphoglyceraldehyde in the dark phase of photosynthesis. The lower value of these parameters may be related to lower plant weight and lower SC in plants growing under LED lamps. In control plants, high correlations between the values of both ψ Ro and φ Ro parameters with the fresh and dry weight of the plants were found. This effect suggests that the quantum efficiency in sunlight with the addition of HPS lamps was higher. In the case of the control plants, a high correlation between the flavonoid content and the energy dissipation (DI_o_/CS_m_) was also found. In plants growing under LED, dispersed energy correlated with the general pool of phenolic compounds. In studied controlled-environment production systems, these compounds probably acted as a photo-protective for the photosynthetic apparatus. In control plants, the parameters of PI, ψ Ro, and φ Ro strongly correlated with the content of chlorophyll index and with the fresh and dry weight of the aboveground parts of common buckwheat. This relationship was not found in plants growing under LED lamps. Under this treatment, PI, ψ Ro, and φ Ro were lower than the control, which could explain the lower FW and DW of aboveground parts of plants.

In our experiment, the plants grown in the greenhouse had a higher chlorophyll index. Chlorophyll biosynthesis requires light and its different qualities regulate the synthesis of photosynthetic pigments^39,40^. Blue light may promote the expression of enzymes that regulate the synthesis of chlorophyll, such as MgCH (magnesium chelatase), FeCH (ferrochelatase), and GluTR (glutamyl-tRNA reductase)^40,41^. However, red light reduces tetrapyrrole precursor 5-aminolevulinic acids, essential for chlorophyll biosynthesis^40,42^, which may explain the lower chlorophyll content in the plants exposed to LED light. In our study, red light seemed to be the more dominant factor regulating chlorophyll biosynthesis than lower light intensity. According to Tripathy and Brown^43^, red light at an intensity of 100 µmol m^–2^ s^–1^ stimulated chlorophyll synthesis, as opposed to a high intensity of 500 µmol m^–2^ s^–1^. In the case of buckwheat plants grown under LEDs with a 53% proportion of red light, i.e., 20% more than sunlight, we observed a significantly lower content of chlorophyll than in the plants grown in the daylight. According to Boardman^44^ and Lichtenthaler et al.^45^ leaves grown at low light intensity have more chlorophyll per unit weight, but lower content calculated per area of leaf surface compared to the leaves grown in the sunlight. In some plant species blue light may increase the value of the chlorophyll a/b ratio ^21^. In turn, Wang et al.^46^ stated that the combined Red and Blue LEDs increased chlorophyll content, photosynthesis rate, leaf number and area, and shoot mass. Muneer et al.^47^ showed that photosynthesis rate, stomatal conductance, and growth depended on Red light/Blue light ratio and these parameters increased with greater blue radiation. These authors stated that for photosynthesis rate and stomatal conductance in lettuce plants the most optimal is when the R/B ratio is 1. In the case of light in the phytotronic chamber, the R/B ratio was 2, in the greenhouse without HPS lamps (most of the day) it was also 2, and with HPS lamps R/B was 3.5. Therefore, the lower chlorophyll content in buckwheat leaves under LEDs may be the result of lower light intensity than a different light spectrum^37^. Although it cannot be excluded that a higher R/B ratio is more optimal for common buckwheat plants.

Landi et al.^21^ reported that typically monochromatic blue light does not have a significant effect on plant biomass compared to plants grown in multispectral light, but there are cases, where plants show an inhibitory effect of blue light on leaf growth, number, and size. However, we found significant differences between FW and DW of the aboveground parts with greater biomass for the plants grown under control conditions. Blue light is known to reduce elongation growth^48–50^, which depends on phytochrome activity affected by background light conditions^51^. It may explain why control plants were taller than those grown under LED spectra. Red light plays a significant role in shoot elongation and plant anatomy through phytochromes^52,53^. In common buckwheat growth, a dominant role of blue light was observed, which resulted in a smaller main shoot height in the plants grown under the LED spectrum. Also, the role of the green region of the spectrum, especially in photosynthesis in the deep layers of the mesophyll and the lower canopy levels, can be large enough. The green light is an influential wave band in supporting photosynthesis in higher plants, especially in leaves with higher chlorophyll index (Sun et al.^54^, Terashima^55^ and Ptushenko^56^), which occurred in our experiment in control plants.

Carbohydrates are products of photosynthesis that regulate the growth and development of leaves^57^. They participate in the synthesis of phenolic compounds that are activated in plant defense mechanisms during environmental stresses^58^. The quantitative and qualitative differences were found for individual soluble carbohydrates content. The monosaccharides (glucose and fructose) content in control plants was significantly higher than in the plants growing under LED. Under this treatment, performance index (PI) correlated with glucose, sucrose, and maltose. Monochromatic red light may induce the accumulation of carbohydrates in leaves due to the inhibition of the translocation of assimilates from source to sink tissue. On the second hand, red light induces the reduction of plant biomass and leaf area, provokes excessive stem elongation, and affects leaf number and chlorophyll content compared to plants grown in white light^21^. In turn, the photosynthetic rate in plants grown under blue LED light is similar to that in plants grown under white light and higher than in plants grown under red light^21^. In our opinion, the lower content of carbohydrates in common buckwheat plants under used LEDs was more related to lower photosynthetic activity than to the light spectrum. Biosynthesis of specified phenylpropanoids is activated during different stages of plant organogenesis in diverse tissues and responds to biotic and abiotic stress factors, e.g., UV-B irradiation^9,59,60^. Biosynthesis of phenylpropanoids requires an adequate flow of carbon via shikimate to the biosynthesis of aromatic amino acids: phenylalanine, tyrosine, and tryptophan. Aromatic amino acids serve as essential precursors to a wide range of secondary plant metabolites. The first step from primary into secondary metabolism is the phenylpropanoid pathway, an intermediate stage in specific branch pathways leading to targeted biosynthesis of, i.a., flavonoids^9,59^. Flavonoids may be included in colourful (flavonols, anthocyanins, and proanthocyanidins) and colourless compounds^61^. Colourless flavonoids, like simple phenolics, absorb mainly UV radiation in the range 335 nm for flavonoids and 280–315 nm for phenolic acids^62^. Some flavonoids absorb other wavelengths, for example, anthocyanins, which absorb the visible light of the solar spectrum^63^. Besides, some flavonoids show antioxidant properties, so they act as scavengers of reactive oxygen species^64^. Moreover, anthocyanins, by absorbing the fraction of yellow, green, and blue wavelengths, may significantly reduce the damage of PSII^21^. Phenolic compounds are known for their protective function against stress factors in harsh environmental conditions such as high temperature, salinity, heavy metal pollution, or ultraviolet radiation^65^. In the present experiment, total phenolic and flavonoid contents were significantly higher in the plants grown under light-emitting diodes than in control ones. The effect of monochromatic red and blue lights on secondary metabolites’ synthesis was studied in the case of *Fagopyrum tataricum*^66,67^. Blue light increased the content of anthocyanin, and the production of zeaxanthin, while reducing the content of total carotenoid compared to the treatment under white light. Lobiuc et al.^68^ reported that rosmarinic and gallic acids synthesis increased under LED blue light. Our results obtained for plants grown under LED light with 25% blue radiation may confirm it. Sytar et al.^69^ reported that direct sunlight with moderate temperature and high UV radiation increased the accumulation of total phenolics, flavonoids, and phenolic acids in lettuce leaves compared to the plants grown in the greenhouse with low UV radiation and high temperature. In our model of experiment, the temperature in the greenhouse was controlled with air-conditioning system and was comparable to the phytotronic chamber condition. We noticed the highest accumulation of total phenolics, flavonoids, and the majority of phenolic acids in buckwheat plants under LED light with the dominant role of red and blue radiation. Anthocyanin accumulation is induced by light and cytokinins^21^. The content of cytokinins in the shadow can be not sufficient to stimulate the accumulation of anthocyanins. In our experiment in phytotronic conditions with lower light intensity than in the greenhouse in the leaves of buckwheat, significantly higher phenolic accumulation was observed, and this was rather an effect of the light spectrum—mostly red and blue radiations, than light intensity. Blue and white light were observed to reinforce the production of phenolic acids^7,70,71^, which explains increased amounts of phenolic acids in the plants exposed to LEDs, with the more vigorous relative intensity of blue peak, than under control light conditions. Phenolic acids can be differentiated into two groups: derivatives of benzoic acid (hydroxybenzoic acids: benzoic, gallic, protocatechuic, *p*-hydroxybenzoic, vanillic, variations of dihydroxybenzoic acid) and derivatives of cinnamic acid (hydroxycinnamic acids: cinnamic, caffeic, *p*-coumaric, ferulic, sinapic)^72^. The amounts of the most studied benzoic acid derivatives increased in the plants grown under LEDs vs. control. Their accumulation, serving as a plant tolerance mechanism against abiotic stress, was stated in many plant species^73^. The genes encoding key enzymes, including HQT (hydroxycinnamoyl-CoA quinate hydroxycinnamoyl transferase) and PAL (phenylalanine ammonia-lyase) families, are upregulated under abiotic stresses, which boosts the biosynthesis of phenolic compounds, such as, e.g., chlorogenic acid. Finally, it may affect plant tolerance to the stress factor. Chlorogenic acid, an ester of trans-hydroxycinnamic acids and quinic acid, is involved in plant response to stress, e.g., pathogens and salinity^74^, and in our experiment, it was the most abundant acid with accumulation increasing more than two times in the leaves of plants grown under LED light vs. controls. The role of the specified light spectrum in photosynthetic efficiency and activity of biosynthetic pathways of valuable metabolites is essential for understanding the mechanisms of plant response to growth conditions, for example, in the greenhouse or phytotronic experiments.

## Conclusions

We concluded that applied solo LED light in phytotronic conditions decreased the biomass growth of common buckwheat compared with solar light supplemented with HPS lamps. However, the used LEDs stimulated the production of phenolic compounds that are considered health-promoting due to their antioxidant properties.

## Materials and methods

### Plant material and growth conditions

**Figure 3:**
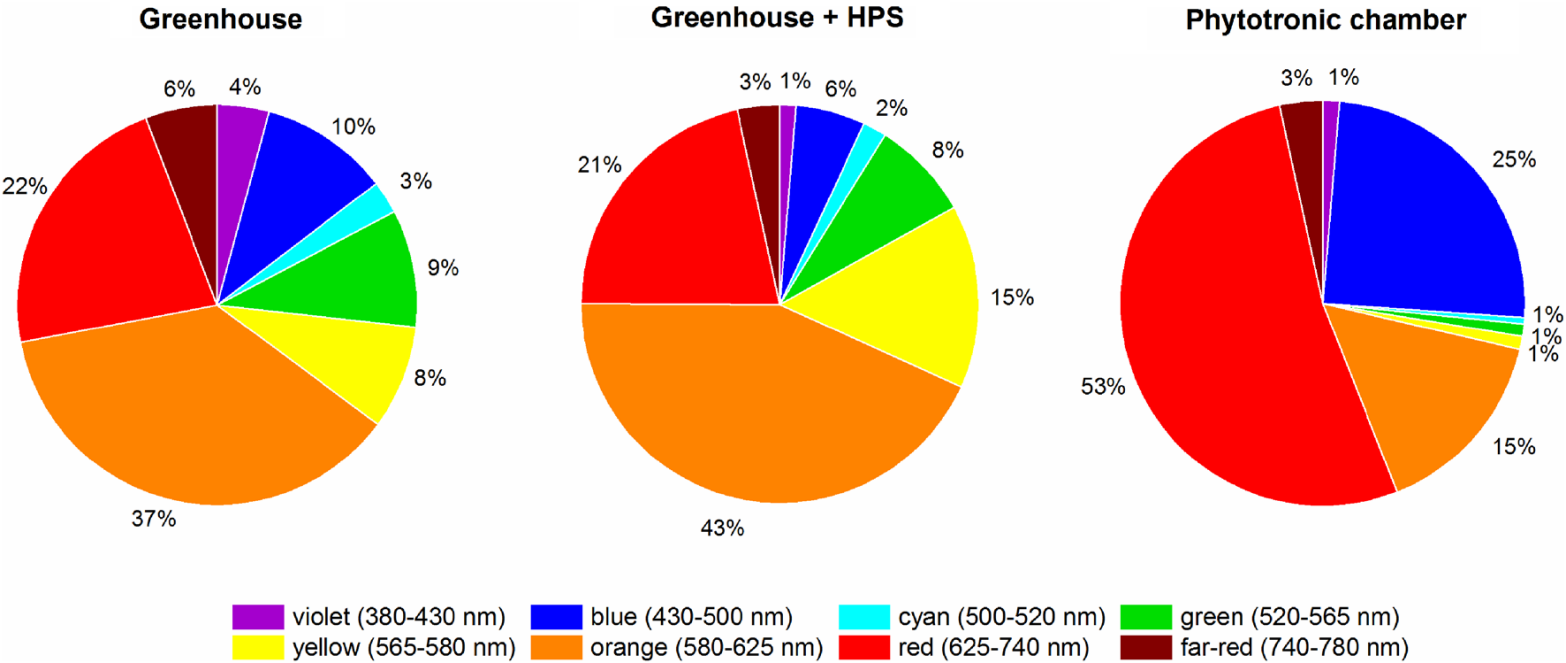
The percentage share of individual colours in the tested spectra. Greenhouse—solar radiation; Greenhouse + HPS—solar radiation supplemented with HPS lamps (High-Pressure Sodium); Phytotronic chamber—solo LED lamps (Light-Emitting diodes). Plants were grown in a greenhouse in the daylight (under 16-h photoperiod) supplemented with HPS lamps’ spectrum from 6.00 to 10.00 a.m., and in a phytotronic chamber with only LED spectrum. Greenhouse and Greenhouse + HPS were presented to demonstrate the influence of HPS spectrum on daylight.

**Figure 4:**
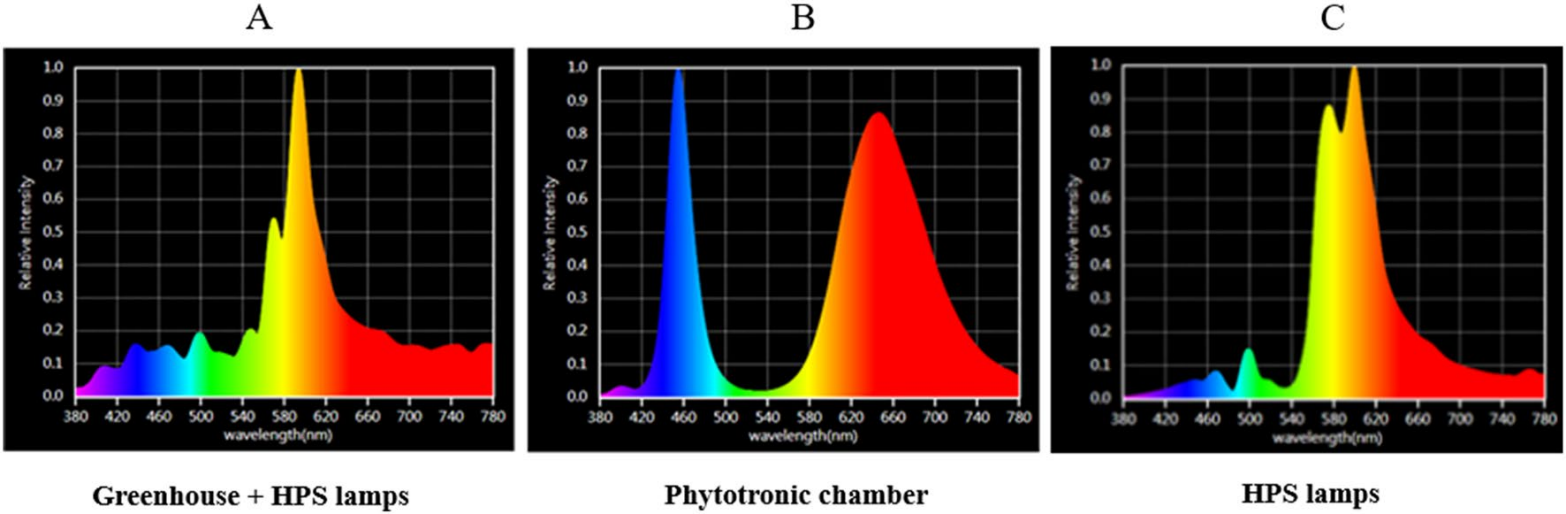
Light spectrum in a greenhouse under solar light supplemented with HPS Agro Phillips lamps (**A**; control), phytotron chamber with LED light (**B**), and the light spectrum emitted by solo HPS (**C**).

The seeds of *F. esculentum* Moench cv. 'Panda' were provided by breeders from Małopolska Plant Breeding in Polanowice, Poland. The experiments were carried out in plants grown under controlled conditions in a phytotron chamber and a greenhouse as a control. The plants were grown in pots (20 × 20 × 25 cm; five plants per pot; ten pots for each treatment) in commercial soil substrate (pH = 5.8) mixed 1:1 (v/v) with perlite. The plants were fertilized once a week with Hoagland medium^75^. The plants were cultivated for eight weeks in August and September 2020 at 25 + 2 °C/22 ± 2 °C day/night and 55–60% humidity. The greenhouse was located at latitude 50° 04′ 10.195″ N and longitude 19° 50′ 44.763″ E, the maximum sunlight intensity on a cloudless day was between 1300 and 1600 μmol (photons) m^–2^ s^–1^ of PPFD (photosynthetic photon flux density), the natural daylight lasted from 5:15 a.m. (sunrise) to 8:20 p.m. (sunset) at the beginning of cultivation and from 5.55 a.m. to 6.20 p.m. at the end of cultivation. In the greenhouse, plants were exposed to solar radiation supplemented with HPS lamps (Fig. 3). The HPS (AGRO Philips) lamps provided an additional 300 μmol (photons) m^–2^ s^–1^ of PPFD from 6:00 a.m. h to 10:00 p.m. under 16 h photoperiod. In the phytotronic chamber with LED light, plants were grown under a 16-h photoperiod with constant light intensity. In greenhouse conditions with daylight, in the period from August to the end of September, the daylight had to be supplemented with an additional light source to ensure the same photoperiod. HPS lamps were turned on in the morning so that the additional light source would not significantly increase the intensity of natural light. The greenhouse was air-conditioned preventing significant temperature fluctuations, which could occur on sunny days as well as under the influence of heat generated by HPS lamps. The used HPS lamps provided a golden yellow light and caused a color shift toward the yellow-orange end of the spectrum (Fig. 4C). The plants in the phytotron chamber were exposed to solo LED light (with blue, red, and addition of UV, green, yellow, orange, and far-red radiation, 200 W Full Spectrum LED Flood Light, Color Temp.: 380–840 nm) (Fig. 3 and 4B) and average 300 μmol (photons) m^–2^ s^–1^ of PPFD from 6:00 a.m. to 10:00 p.m. Composition of the solar light, and HPS lamps gave more green, yellow and orange waves than the LED spectrum (Fig. 3 and 4A). DLI (daily light integral) value was calculated to compare two studied sources of light: LED light in isolated chamber and greenhouse with daylight supplemented for 4 h with HPSs light. This parameter describes the number of photosynthetically active photons (photons in the PAR range) that are delivered to a specific area (1 m^2^) over a 24 h-period. DLI is calculated by measuring the photosynthetic photon flux density (PPFD) in μmol m^−2^ s^−1^ for a specific area as it changes throughout the day^76–78^. DLI = PPFD [μmol m^−2^ s^−1^] × (3600 × photoperiod)/1,000,000 [mol m^−2^ day^−1^]. The DLI value for the greenhouse with HPS was range from 10 mol m^−2^ day^−1^ during cloudy days to 38.5 mol m^−2^ day^−1^ in sunny days, while for the phytotronic chamber it was 23 mol m^−2^ day^−1^. The calculation suggests that in aproximately 9 h a day light under LEDs was lower than in the greenhouse.

The light spectrum was recorded after germination at the top of 2-week-old seedlings by a spectrometer Lighting Passport Pro (Asensetek, Taiwan) with Spectrum Genius Cloud (Taiwan) software. The range of individual sub-regions of the visible light was selected according to Malacara^79^.

### Plant growth measurements.

The measurements of the studied parameters were executed in the leaves (fully developed young leaves, third in the order from the top inflorescences) of 8-week-old plants at the stage of full flowering. Buckwheat blooming lasts throughout the entire vegetative phase. The measurements included shoot (whole aboveground parts), fresh weight (FW), and dry weight (DW), leaf area (LA), internode amount, and main stem height, and were done in ten replicates. Plant samples were transferred into a drying oven at 80 °C for 48 h to obtain dry weight. Every plant's LA (cm^2^) was measured with an LA meter (CI-202 Laser Area Meter, CID Bio-Science, WA, USA). The measurements were done for ten plants. The study was in compliance with relevant institutional, national, and international guidelines and legislation.

### Chlorophyll *a* fluorescence (ChlF)

Before the measurements, the LED-light source of a fluorimeter was calibrated using an SQS light meter (Hansatech Ltd., King's Lynn, UK). Excitation irradiance intensity was 3000 [μmol m^−2^ s^−1^] (peak at 650 nm). Measurements were taken after 30 min of the leaf adaptation to darkness (clips with a 4 mm diameter hole). Changes in fluorescence were recorded during irradiation between 10 μs and 1 s. During the initial 2 ms, the data were collected every 10 μs with 12-bit resolution. After this period, the frequency of measurements was reduced automatically. The data were used to calculate the following parameters based on the theory of energy flow in PSII and the JIP test^80,81^: ABS/CS_m_*—*energy absorption by antennas, TR_o_/CS_m_*—*excitation energy trapped in PSII, ET_o_/CS_m_*—*the energy used for electron transport, DI_o_/CS_m_*—*energy dissipation from PSII, RC/CS_m_*—*number of active reaction centers, PI*—*performance index of PSII, ψ Ro*—*probability, at time 0, that a trapped exciton moves an electron into the electron transport chain beyond Q_A_^−^, δ Ro*—*efficiency with which an electron can move from the reduced intersystem of electron acceptors to the PSI end electron acceptors, φ Ro*—*quantum yield of electron transport from Q_A_^−^ to the PSI end electron acceptors. The measurements included twenty plants per treatment.

### Chlorophyll index

Leaf chlorophyll index was measured non-destructively with a hand-held chlorophyll meter (CL-01 Hansatech Instruments, King's Lynn, UK) in twenty repetitions for each treatment. We analyzed the third, fully developed leaf from the top inflorescence in eight-week-old plants. The measurements were done for twenty plants.

## Biochemical analyses

### Sample preparation for biochemical analyses

The samples involved fully developed young leaves, third in the order from the top inflorescences. The samples were collected, frozen in liquid nitrogen, and then lyophilized (LGA05, MLW, Leipzig, Germany, upgraded by JWE, Warsaw, Poland). Afterward, they were pulverized (MM400, Retsch, Haan, Germany), and the material was used for further analyses. Each sample for the biochemical analysis was 15 mg of DM.

### Soluble sugar profile

Sugars were estimated using the method reported by Hura et al.^82^. The samples were extracted with ultra-pure water by shaking for 15 min at 30 Hz (MM 400, Retsch, Haan, Germany) and centrifuged for 5 min at 21,000×*g* (Universal 32R, Hettich, Germany). Then, the supernatant was collected, diluted with acetonitrile 1:1 (v/v), filtered (0.22 µm nylon membrane, Costar Spin-X, Corning, USA), and analyzed by high-performance liquid chromatography (HPLC) using an Agilent 1200 binary system (Agilent, Wolbrum, Germany) coupled with ESA Coulochem II electrochemical detector (ESA, Chelmsford, MA, USA). An RCX-10, 7 µm, 250 × 4.1 mm column (Hamilton, Reno, NV, USA) in a 75 mM NaOH solution gradient mode, and 500 mM sodium acetate in 75 mM NaOH solution at 1.5 ml/min, was used. Pulsed amperometric detection was employed on a gold electrode. Further technical details are given by Hura et al.^82^. The analysis was performed in three biological replicates.

### Total phenolic content

The total soluble phenolic content was estimated according to the method reported by Singleton et al.^83^ with minor modifications. The extract was diluted in deionized water (0.5 cm^3^) with the addition of Folin–Ciocalteu reagent (0.2 cm^3^). After 10 min of incubation, saturated Na_2_CO_3_ (0.7 cm^3^) was added. Then, after a 2 h incubation, the samples were mixed and transferred into 96-well plates. The absorbance at 765 nm was read (Synergy II). Gallic acid was used as a standard. The analysis was carried out in three biological replicates.

### Phenolic acid content

Phenolic acids were estimated according to Hura et al.^82^. The samples were extracted in an organic buffer (methanol/water/formic acid 15/4/1 /v/v/v). After evaporation under nitrogen stream (TurboVap LV), the residue was solubilized in 3% methanol in 1 M formic acid before clean-up on Discovery DPA-6S SPE cartridges (1 ml, 50 mg, Supelco). The eluate was evaporated under N_2_, reconstituted in 250 µl of methanol, and analyzed on Agilent Infinity 1260 UHPLC (Ultra-High-Performance Liquid Chromatography) with a fluorescence detector (FLD). The phenolic acids were separated on Zorbax Eclipse Plus Phenyl-Hexyl 3.5 µm 3.0 mm × 100 mm column (Agilent Technologies) under a linear gradient of 2% (v/v) formic acid aqueous solution versus methanol. Excitation and emission wavelengths were dynamically adjusted. Technical details are provided in Gołębiowska-Pikania et al.^84^. The analysis was done in three biological replicates.

### Total flavonoid content

The analyses of total flavonoid content in the extracts were done according to Ramos et al.^85^, as reported by Klimek-Szczykutowicz et al.^86^. The samples were extracted with methanol, then 100 µl of the centrifuged extract were mixed with 40 µl of 10% AlCl_3_, filled with 5% acetic acid to a final volume of 1000 µl, and incubated for 20 min. Then the absorbance at 425 nm was read in 96-well plate format (Synergy II). Quercetin was used as a reference. The analysis was carried out in three biological replicates.

### Statistical analysis

One-way analysis of variance (ANOVA) was performed using STATISTICA 13 package (Statsoft, Tulsa, OK, USA). Significance of differences between means obtained in the studied light conditions was marked with stars according to the Student's *t* test: *p < 0.05; **p < 0.01; ***p < 0.001. Values represent means ± SE (standard error). Pearson’s correlation coefficients were assumed as statistically significant at *p* < 0.05. The used parameters taken for correlation analysis referred to the same plants.

## Supplementary Information

Supplementary Table S1.
