# Supplementary material for: Photosynthetic efficiency, growth and secondary metabolism of common buckwheat (Fagopyrum esculentum Moench) in different controlled-environment production systems
Source: Sci Rep. 2022 Jan 7;12:257. doi: 10.1038/s41598-021-04134-6 (PMC8741924; doi:10.1038/s41598-021-04134-6)
Supplement: Supplementary file 1 — Supplementary Table S1. [file 41598_2021_4134_MOESM1_ESM.docx]

| Genome | Treatment | No. | PI | Fv/Fm | ABS/CS_m_ | TRo/CS_m_ | ETo/CS_m_ | DIo/CS_m_ | RC/CS_m_ | ẟ Ro | φ Ro | ψ Ro | Fv/F0 |
| --- | --- | --- | --- | --- | --- | --- | --- | --- | --- | --- | --- | --- | --- |
| Panda | Control | 1 | 2,022 | 0,839 | 1480 | 1242 | 578 | 238 | 658,696 | 0,363 | 0,142 | 0,169 | 5,218 |
| Panda | Control | 2 | 1,966 | 0,833 | 1524 | 1270 | 604 | 254 | 660,797 | 0,318 | 0,126 | 0,151 | 5,000 |
| Panda | Control | 3 | 1,734 | 0,838 | 1569 | 1315 | 582 | 254 | 662,016 | 0,393 | 0,146 | 0,174 | 5,177 |
| Panda | Control | 4 | 1,523 | 0,825 | 1492 | 1231 | 534 | 261 | 629,037 | 0,288 | 0,103 | 0,125 | 4,716 |
| Panda | Control | 5 | 2,299 | 0,845 | 1580 | 1335 | 667 | 245 | 667,500 | 0,303 | 0,128 | 0,151 | 5,449 |
| Panda | Control | 6 | 0,903 | 0,821 | 1486 | 1220 | 399 | 266 | 601,935 | 0,391 | 0,105 | 0,128 | 4,586 |
| Panda | Control | 7 | 2,828 | 0,844 | 1491 | 1259 | 674 | 232 | 674,464 | 0,323 | 0,146 | 0,173 | 5,427 |
| Panda | Control | 8 | 1,826 | 0,833 | 1524 | 1270 | 582 | 254 | 657,952 | 0,405 | 0,155 | 0,186 | 5,000 |
| Panda | Control | 9 | 2,175 | 0,843 | 1480 | 1247 | 625 | 233 | 598,483 | 0,291 | 0,123 | 0,146 | 5,352 |
| Panda | Control | 10 | 3,678 | 0,849 | 1472 | 1250 | 757 | 222 | 626,270 | 0,365 | 0,188 | 0,221 | 5,631 |
| Panda | Control | 11 | 1,319 | 0,819 | 1454 | 1191 | 485 | 263 | 616,456 | 0,433 | 0,144 | 0,176 | 4,529 |
| Panda | Control | 12 | 4,039 | 0,838 | 1384 | 1160 | 737 | 224 | 619,545 | 0,370 | 0,197 | 0,235 | 5,179 |
| Panda | Control | 13 | 1,226 | 0,815 | 1447 | 1180 | 479 | 267 | 587,486 | 0,468 | 0,155 | 0,190 | 4,419 |
| Panda | Control | 14 | 2,479 | 0,837 | 1515 | 1268 | 660 | 247 | 673,902 | 0,308 | 0,134 | 0,160 | 5,134 |
| Panda | Control | 15 | 1,856 | 0,828 | 1469 | 1217 | 579 | 252 | 622,152 | 0,408 | 0,161 | 0,194 | 4,829 |
| Panda | Control | 16 | 1,297 | 0,825 | 1425 | 1175 | 474 | 250 | 581,691 | 0,401 | 0,133 | 0,162 | 4,700 |
| Panda | Control | 17 | 2,008 | 0,836 | 1542 | 1289 | 628 | 253 | 639,661 | 0,346 | 0,141 | 0,168 | 5,095 |
| Panda | Control | 18 | 2,725 | 0,845 | 1469 | 1242 | 662 | 227 | 640,890 | 0,307 | 0,138 | 0,163 | 5,471 |
| Panda | Control | 19 | 1,5 | 0,83 | 1533 | 1273 | 546 | 260 | 625,318 | 0,355 | 0,127 | 0,152 | 4,896 |
| Panda | Control | 20 | 2,203 | 0,838 | 1505 | 1261 | 639 | 244 | 624,476 | 0,344 | 0,146 | 0,174 | 5,168 |
| Panda | LED | 1 | 1,941 | 0,839 | 1488 | 1249 | 570 | 239 | 658,440 | 0,430 | 0,165 | 0,196 | 5,226 |
| Panda | LED | 2 | 1,521 | 0,826 | 1616 | 1335 | 583 | 281 | 667,500 | 0,403 | 0,145 | 0,176 | 4,751 |
| Panda | LED | 3 | 2,214 | 0,848 | 1600 | 1356 | 643 | 244 | 706,746 | 0,342 | 0,138 | 0,162 | 5,557 |
| Panda | LED | 4 | 0,24 | 0,757 | 1504 | 1138 | 201 | 366 | 540,723 | 0,522 | 0,070 | 0,092 | 3,109 |
| Panda | LED | 5 | 1,736 | 0,823 | 1466 | 1206 | 555 | 260 | 643,530 | 0,362 | 0,137 | 0,167 | 4,638 |
| Panda | LED | 6 | 2,92 | 0,84 | 1622 | 1363 | 721 | 259 | 801,324 | 0,340 | 0,151 | 0,180 | 5,263 |
| Panda | LED | 7 | 1,262 | 0,838 | 1522 | 1276 | 484 | 246 | 605,871 | 0,306 | 0,097 | 0,116 | 5,187 |
| Panda | LED | 8 | 0,899 | 0,804 | 1415 | 1138 | 406 | 277 | 558,322 | 0,271 | 0,078 | 0,097 | 4,108 |
| Panda | LED | 9 | 1,637 | 0,833 | 1603 | 1336 | 592 | 267 | 659,141 | 0,326 | 0,120 | 0,144 | 5,004 |
| Panda | LED | 10 | 1,206 | 0,824 | 1684 | 1387 | 548 | 297 | 665,728 | 0,310 | 0,101 | 0,123 | 4,670 |
| Panda | LED | 11 | 2,758 | 0,846 | 1690 | 1430 | 742 | 260 | 785,815 | 0,317 | 0,139 | 0,164 | 5,500 |
| Panda | LED | 12 | 1,497 | 0,836 | 1632 | 1364 | 584 | 268 | 640,916 | 0,396 | 0,142 | 0,169 | 5,090 |
| Panda | LED | 13 | 2,516 | 0,862 | 1793 | 1546 | 759 | 247 | 747,360 | 0,213 | 0,090 | 0,105 | 6,259 |
| Panda | LED | 14 | 0,835 | 0,812 | 1434 | 1165 | 373 | 269 | 586,947 | 0,523 | 0,136 | 0,167 | 4,331 |
| Panda | LED | 15 | 2,859 | 0,852 | 1726 | 1471 | 777 | 255 | 764,127 | 0,328 | 0,148 | 0,173 | 5,769 |
| Panda | LED | 16 | 0,964 | 0,769 | 1781 | 1370 | 591 | 411 | 678,899 | 0,333 | 0,111 | 0,144 | 3,333 |
| Panda | LED | 17 | 1,564 | 0,841 | 1595 | 1341 | 596 | 254 | 590,452 | 0,315 | 0,118 | 0,140 | 5,280 |
| Panda | LED | 18 | 0,355 | 0,749 | 1548 | 1160 | 333 | 388 | 455,951 | 0,435 | 0,094 | 0,125 | 2,990 |
| Panda | LED | 19 | 0,388 | 0,758 | 1561 | 1184 | 339 | 377 | 481,000 | 0,451 | 0,098 | 0,129 | 3,141 |
| Panda | LED | 20 | 1,195 | 0,83 | 1550 | 1286 | 512 | 264 | 574,691 | 0,307 | 0,101 | 0,122 | 4,871 |

Supplementary Table 1. The raw data for changes in the kinetics of chlorophyll *a* fluorescence in common buckwheat plants of cv. ‘Panda’ grown under different controlled-environment production systems. Control – solar light supplemented with HPS (High-Pressure Sodium) lamps; LED (Light-Emitting Diodes), *n* = 20. PI – performance index of PSII, Fv/Fm – maximal quantum yield of PSII photochemistry, ABS/CS_m_ – energy absorption by antennas, TRo/CS_m_ – excitation energy trapped in PSII, ETo/CS_m_ – the energy used for electron transport, DIo/CS_m_ – energy dissipation from PSII, RC/CS_m_ – number of active reaction centers, ẟ Ro – efficiency with which an electron can move from the reduced intersystem of electron acceptors to the PSI end electron acceptors, φ Ro – quantum yield of electron transport from Q_A_^−^ to the PSI end electron acceptors, ψ Ro – probability, at time 0, that a trapped exciton moves an electron into the electron transport chain beyond Q_A_ ^−^, Fv/F0 – maximum efficiency of water-splitting reaction of the donor side of PSII.
